# No Significant Difference between Plasma miRNAs and Plasma-Derived Exosomal miRNAs from Healthy People

**DOI:** 10.1155/2017/1304816

**Published:** 2017-06-01

**Authors:** Fei Tian, Yanting Shen, Zhenzhu Chen, Rui Li, Qinyu Ge

**Affiliations:** ^1^Research Center for Learning Science, Southeast University, Sipailou No. 2, Nanjing, Jiangsu 210096, China; ^2^State Key Lab of Bioelectronics, Southeast University, Nanjing 210096, China

## Abstract

**Background:**

MicroRNAs (miRNAs) are small noncoding RNAs that are involved in many biological regulation processes. Studies have reported that miRNAs are enriched in human plasma and plasma-derived exosome as novel diagnostic biomarkers. The aim of this study was to determine whether the miRNA expression levels are different between plasma and plasma-derived exosome.

**Methods:**

We sequenced and quantified the miRNAs in plasma and exosome from healthy blood samples and validated three miRNAs in the two groups of lung cancer samples by qRT-PCR.

**Results:**

The sequencing results showed that only several of miRNAs were differential, while the qRT-PCR further validated that most of them did not have the consistent differences. However, the levels of two upregulated miRNAs (miR-181b-5p and miR-21-5p) in lung cancer were significantly higher in exosomes than plasma.

**Conclusions:**

To the best of our knowledge, this study is the first to compare the expression levels of miRNAs between plasma and exosome in healthy blood samples. Our data suggested that the miRNA levels were similar in the two parts of the healthy people, whereas the two onco-miRNAs were significantly enriched in the exosome of lung cancer patients.

## 1. Introduction

MicroRNA (miRNA) is a kind of small endogenous noncoding RNA of about 18–25 nucleotides [1]. Lee et al. first found that the* C. elegans* gene lin-4 encoded small RNAs (miRNAs) in 1993 [2]. Mature miRNAs are highly conserved RNAs, processed from initial transcript by nuclease Drosha and Dicer. MiRNAs can perform its regulation function to inhibit or degrade mRNAs by hybridizing to complementary sequences in the 3′-untranslated region of target [3, 4]. Up to date, miRNAs have been found in various organisms and participate in a lot of biological processes [5, 6].

Plasma is an ideal biological sample for the detection of many diseases because of its rich resource, convenient access, and noninvasion. One of the first studies detecting and characterizing plasma miRNAs was reported by Chim et al. who demonstrated the existence of placental miRNAs in maternal plasma [7]. Mitchell et al. validated that plasma miRNAs were present in a remarkably stable form that was protected from endogenous RNase activity [8]. Now, plasma miRNAs are widely studied in the diagnosis, prognosis, and treatment of various diseases including cancers [9, 10].

Exosomes are classically described as 50–100 nm vesicles of endocytic origin that released into the extracellular environment on fusion of multivesicular bodies with the plasma membrane [11]. Diverse cells have the capacity to release exosomes, including B cells, T cells, epithelial cells, and tumor cells [12–15]. Studies reported that circulating exosomes were present in human blood plasma [16, 17]. More interestingly, miRNAs have been reported to present in exosome preparations, which can be delivered between cells as a mechanism of genetic exchange [18]. Moreover, exosomal miRNAs have been a subject of great interest as novel biomarkers in many diseases [19–21]. Our former study showed that exosomal miRNAs had the extra stability under different storage conditions [22].

Both plasma and exosome miRNAs receive much concern as diagnostic markers in disease-related studies in recent years. However, few studies determine whether the miRNA expression levels are different between plasma and plasma-derived exosome. In this study, we detected the miRNA levels in 10 paired plasma and exosome samples from healthy people by Illumina Hiseq2500. The overall results illustrated that the expression levels of miRNAs were similar in paired plasma and exosome samples from healthy people. Nevertheless, the levels of two onco-miRNAs were significantly different between plasma and exosome from lung cancer (LC) patients.

## 2. Materials and Methods

### 2.1. Sample Collection, Exosome Isolation, and RNA Extraction

We collected 30 whole blood samples from 30 healthy volunteers recruited for this study. All individuals conducted physical examinations to ensure the healthy state in Anqing Municipal Hospital. And 10 blood samples from early stage LC patients were obtained in Jiangsu Province Hospital with informed consent. Demographic data and clinical information for all subjects are presented in Tables 1 and 2. Plasma was isolated from 4 mL blood for subsequent RNA and exosome isolation.

Exosome was precipitated from 1 mL plasma for immediate RNA isolation according to the protocol of ExoQuick™ Exosome Precipitation Solution (SBI, USA), and the supernatant after exosome precipitating was reserved. Circulating RNA was isolated from 1 mL remaining plasma and the supernatant by miRNeasy Serum/Plasma Kit (Qiagen, Germany). The quantity and quality of all obtained RNAs were measured by 2100 Bioanalyzer (Agilent, USA). The total amount of RNA was more than 10 *μ*g, and the RNA Integrity Number (RIN) was more than 7.

### 2.2. Small RNA Sequencing for Healthy Samples and Bioinformation Analysis

Plasma RNA, exosome RNA, and supernatant RNA were respectively pooled from 10 healthy samples for small RNA library preparation and Illumina Hiseq2500 sequencing as described in our previous study [23]. The obtained reads were mapped to the corresponding database. To compare miRNA expression across databases, the total copy number of each sample was normalized to 1,000,000 [24]; *p* value was calculated by Benjamini-Hochberg method.

### 2.3. Quantitative Validation by qRT-PCR

We performed quantitative RT-PCR (qRT-PCR) to validate the miRNA levels in another 20 paired plasma and exosome samples from healthy individuals. And 3 miRNAs in our previous LC study (miR-181b-5p, miR-21-5p, and miR-486-5p) were analyzed for expression differences between plasma and exosome [23]. The reverse transcription reaction was carried out with PrimeScript™ II reverse transcriptase (Takara, Japan). The quantitative PCR amplification was performed with SYBR Premix Ex Taq™ (Takara, Japan) and the reaction was incubated in ABI 7500 PCR system (ABI, USA). U6 snRNA was used as the internal reference of miRNA. All primers used in this study were listed in Supplemental Table 1 in Supplementary Material available online at https://doi.org/10.1155/2017/1304816.

### 2.4. Statistical Analysis

We adopted the log10 method to transform the normalized reads and the log2 method to transform the fold change. The average Ct for each triplicate from qRT-PCR was calculated, and fold change in miRNA expression was calculated by ΔΔCt normalized with ΔCt = AvgCt_miRNA_ − AvgCt_U6_ [25]. Comparison of two groups was performed using the *t*-test; *p* value < 0.05 was considered to indicate statistically significant difference.

## 3. Results

### 3.1. The Expression Levels of MiRNAs in Plasma and Plasma-Derived Exosome from Healthy People

**Figure 1:**
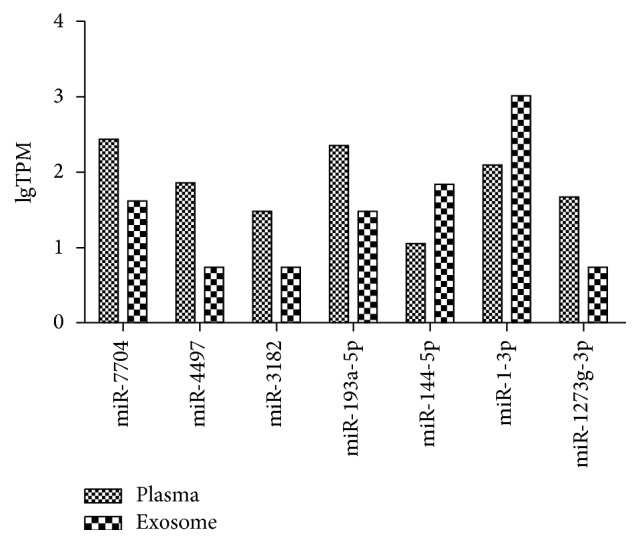
The 7 differential miRNAs between plasma and exosome in the healthy group by sequencing. The *y*-axis meant the value of TPM (transcripts per million) as log10. Among these miRNAs, 5 miRNAs were significantly upregulated in the plasma compared to exosome, and 2 miRNAs were downregulated (*p* value < 0.05).

The paired plasma, exosome, and supernatant miRNAs were characterized based on Hiseq2500 platform. About 10 M reads of each mixed sample were obtained. However, only 7 miRNAs had significant differences with log_2_ (fold change) > 1 or <−1 and *p* value < 0.05 between plasma and exosome. Among them, 5 miRNAs were upregulated in the plasma compared to exosome, and 2 miRNAs were downregulated (Figure 1, Table 3). In addition, 10 miRNAs had significant differences between plasma and supernatant and 2 miRNAs were differential in exosome compared to the supernatant (Supplemental Figure 1, Supplemental Table 2).

### 3.2. The qRT-PCR Results in the Healthy Samples

**Figure 2:**
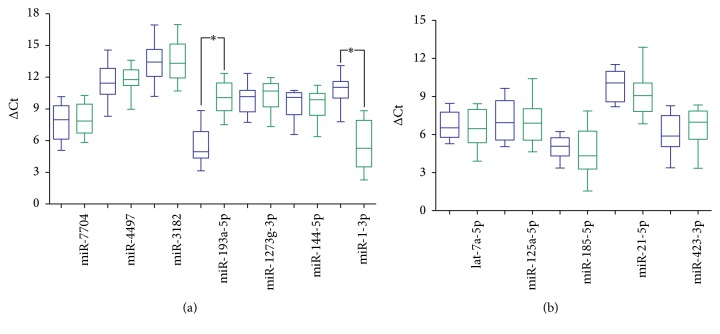
The quantification results of plasma miRNAs and exosome miRNAs in the healthy samples. (a) The 7 differential miRNAs from sequencing: only 2 miRNAs (miR-193a-5p, miR-1-3p) had significant differences between plasma (blue) and exosome (green) (*p* value < 0.05). (b) The 5 nondifferential miRNAs: no differences in the qPCR results. The *y*-axis meant the value of ΔCt = AvgCt_miRNA_ − AvgCt_U6_. The asterisk “*∗*” means the *p* value is less than 0.05 (<0.05).

To validate the expression levels of miRNAs of the healthy samples, 5 randomly selected miRNAs which did not reveal the differences in the sequencing and 7 differential miRNAs between plasma and exosome were chosen to perform qRT-PCR in 20 extra paired healthy samples. The quantification results showed that only 2 miRNAs of the differential miRNAs group had similar expression differences with sequencing results (Figure 2). The supernatant group did not show the consistent differences whether compared to the plasma or compared to the exosome (Supplemental Figure 2).

### 3.3. The qRT-PCR Results in the Lung Cancer Samples

**Figure 3:**
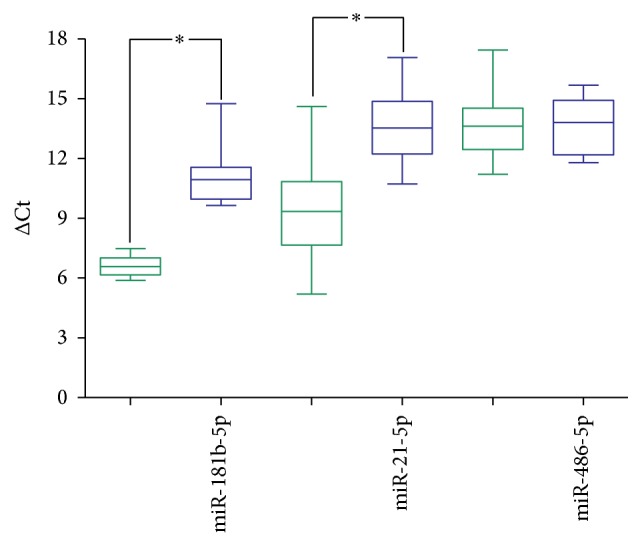
The quantification results of 3 miRNAs between plasma and exosome in the lung cancer samples. MiR-181b-5p and miR-21-5p were significantly upregulated in exosome (green) than in plasma (blue, *p* value < 0.05), but miR-486-5p did not reveal the difference in these two groups. The *y*-axis meant the value of ΔCt = AvgCt_miRNA_ − AvgCt_U6_. The asterisk “*∗*” means the *p* value is less than 0.05 (<0.05).

In addition, our former study revealed that miR-181b-5p and miR-21-5p were upregulated and miR-486-5p was downregulated in nonsmall cell lung cancer (NSCLC) tissue and serum samples compared to the normal controls [23]. In the study, we intended to quantify these 3 miRNAs to determine whether their expression levels were also similar between plasma and exosome from NSCLC blood samples. However, miR-181b-5p and miR-21-5p levels were significantly higher in exosome than in plasma, and miR-486-5p did not reveal the significant bias in the two fractions (Figure 3).

## 4. Discussion

Although plasma miRNAs and exosome miRNAs are widely studied as diagnostic or therapeutic biomarkers in many disease-related researches, few people concern the expression relationship of miRNAs in circulating plasma and plasma-derived exosome. However, similar studies could be found in urinary miRNAs. Zavesky et al. compared the urinary exosome miRNAs and supernatant miRNAs and found that these two fractions had different miRNA expressions in gynaecological cancers [26]. Some studies focused on revealing how the miRNAs existed in circulating conditions or exosome. Arroyo et al. found potentially 90% of miRNAs in the circulation were present in a non-membrane-bound form, and circulating miRNAs cofractionated with Argonaute2 complexes rather than with vesicles [27]. Chevillet et al. found that most individual exosomes contained a small minority of the miRNA content of plasma [28]. However, we cannot ignore the existence of exosome miRNAs as described in many exosome-related studies [18, 29].

In this study, we intended to characterize the expression differences in plasma miRNAs and exosome miRNAs from whole blood samples. Although several miRNAs from healthy people were differential in the sequencing results, the qPCR validation showed that most of them did not have significant differences. Therefore, the overall results suggested that the expression levels of miRNAs were similar in paired plasma and exosome samples in healthy people. We inferred a balanced state between circulating plasma and membrane-bound exosome in healthy individuals. We need to consider whether or not to split exosome more thoroughly when extracting plasma miRNAs in future studies. Based on the hypothesis exosome has equal quantity of miRNAs with plasma; exosome miRNAs can be used to validate the plasma miRNA levels in healthy samples. In addition, our study focused on miRNAs; it is possible that other classes of RNA commonly measured in such biomarker studies, such as mRNAs, may be different in these two samples.

Lung cancer (LC) is the leading cause of cancer deaths in China [30]. Patients with LC are frequently diagnosed at advanced stages, resulting in a low 5-year survival rate, only about 18% [31]. Therefore, the early diagnosis of LC, especially molecular biomarker diagnosis, is important in recent years. In this study, we found that the levels of three miRNAs dysregulated in LC were significantly different between exosome-free plasma and exosome. Exosome-mediated miRNA transfer between cells has been proposed to be a mechanism for intercellular signaling and communication [18, 32–34]. We consider the possibility that miRNAs are selected to be transported via exosomes especially in unhealthy status. The oncogenes (miR-181b-5p, miR-21-5p) were selectively enriched in exosomes rather than the tumor suppressor genes (miR-486-5p). The levels of onco-miRNAs (miR-181b-5p, miR-21-5p) in exosomes were higher than in body fluid and were more similar to the lung cancerous tissue because exosomes were secreted by tissue cells. Therefore, it is necessary to detect exosome miRNAs separately in cancer.

In conclusion, we characterized the miRNA levels in plasma and plasma-derived exosome from healthy and lung cancer individuals, respectively. This study is the first to compare the expression levels of miRNAs between plasma and exosome. Our data suggested that the miRNA levels were similar in the two parts of the healthy case, whereas the two onco-miRNAs were significantly enriched in the exosome of lung cancer case. This study may provide valuable insight into plasma or exosome miRNA related research.

## Supplementary Material

Supplemental Figure 1: The 12 differential miRNAs in the supernatant compared to plasma or exosome in the healthy group by sequencing. Supplemental Figure 2: The quantification results of 5 supernatant miRNAs in the healthy samples. Supplemental Table 1: The primer sequence used in the qRT-PCR assay. Supplemental Table 2: The differentially expressed miRNAs in plasma vs supernatant and in supernatant vs exosome by sequencing.

## Figures and Tables

**Table 1:** Volunteers' demographic data.

Number	Age	Race	Sex
30	27.2 ± 9.8	CHN	15 M, 15 F

**Table 2:** Lung cancer patients' clinical data.

Number	Gender	Age	Tumor subtype	Tumor stage
1	M	45	SCC	IA
2	M	55	SCC	IIA
3	F	52	SCC	IA
4	M	57	AC	IA
5	M	60	AC	IA
6	F	47	SCC	IA
7	M	43	AC	IA
8	F	63	AC	IIA
9	F	55	AC	IB
10	F	59	SCC	IA

^*∗*^SCC: squamous cell carcinoma; AC: adenocarcinoma.

**Table 3:** The differentially expressed miRNAs in plasma versus exosome by sequencing.

miRNA	log_2_ (fold change)	*p* value
hsa-miR-7704	2.71	0.0486
hsa-miR-4497	3.70	0.0117
hsa-miR-3182	2.45	0.0377
hsa-miR-193a-5p	2.89	0.0221
hsa-miR-1273g-3p	3.09	0.0090
hsa-miR-144-5p	−2.61	0.0470
hsa-miR-1-3p	−3.04	0.0359

## Abbreviations

miRNA: microRNA
LC: Lung cancer
qRT-PCR: Quantitative RT-PCR
NSCLC: Nonsmall cell lung cancer.

## References

[1] Bartel D. P. (2004). MicroRNAs: genomics, biogenesis, mechanism, and function. *Cell*.

[2] Lee R. C., Feinbaum R. L., Ambros V. (1993). The C. elegans heterochronic gene *lin*-4 encodes small RNAs with antisense complementarity to *lin*-14. *Cell*.

[3] Bartel D. P. (2009). MicroRNAs: target recognition and regulatory functions. *Cell*.

[4] Valencia-Sanchez M. A., Liu J., Hannon G. J., Parker R. (2006). Control of translation and mRNA degradation by miRNAs and siRNAs. *Genes and Development*.

[5] Lagos-Quintana M., Rauhut R., Yalcin A., Meyer J., Lendeckel W., Tuschl T. (2002). Identification of tissue-specific MicroRNAs from mouse. *Current Biology*.

[6] Krol J., Loedige I., Filipowicz W. (2010). The widespread regulation of microRNA biogenesis, function and decay. *Nature Reviews Genetics*.

[7] Chim S. S. C., Shing T. K. F., Hung E. C. W. (2008). Detection and characterization of placental microRNAs in maternal plasma. *Clinical Chemistry*.

[8] Mitchell P. S., Parkin R. K., Kroh E. M. (2008). Circulating microRNAs as stable blood-based markers for cancer detection. *Proceedings of the National Academy of Sciences of the United States of America*.

[9] Zampetaki A., Kiechl S., Drozdov I. (2010). Plasma MicroRNA profiling reveals loss of endothelial MiR-126 and other MicroRNAs in type 2 diabetes. *Circulation Research*.

[10] Kosaka N., Iguchi H., Ochiya T. (2010). Circulating microRNA in body fluid: a new potential biomarker for cancer diagnosis and prognosis. *Cancer Science*.

[11] van Niel G., Porto-Carreiro I., Simoes S., Raposo G. (2006). Exosomes: a common pathway for a specialized function. *Journal of Biochemistry*.

[12] Raposo G., Nijman H. W., Stoorvogel W. (1996). B lymphocytes secrete antigen-presenting vesicles. *Journal of Experimental Medicine*.

[13] Mittelbrunn M., Gutiérrez-Vázquez C., Villarroya-Beltri C. (2011). Unidirectional transfer of microRNA-loaded exosomes from T cells to antigen-presenting cells. *Nature Communications*.

[14] van Niel G., Mallegol J., Bevilacqua C. (2003). Intestinal epithelial exosomes carry MHC class II/peptides able to inform the immune system in mice. *Gut*.

[15] Wolfers J., Lozier A., Raposo G. (2001). Tumor-derived exosomes are a source of shared tumor rejection antigens for CTL cross-priming. *Nature Medicine*.

[16] Caby M. P., Lankar D., Vincendeau-Scherrer C., Raposo G., Bonnerot C. (2005). Exosomal-like vesicles are present in human blood plasma. *International Immunology*.

[17] Huang X., Yuan T., Tschannen M. (2013). Characterization of human plasma-derived exosomal RNAs by deep sequencing. *BMC Genomics*.

[18] Valadi H., Ekström K., Bossios A., Sjöstrand M., Lee J. J., Lötvall J. O. (2007). Exosome-mediated transfer of mRNAs and microRNAs is a novel mechanism of genetic exchange between cells. *Nature Cell Biology*.

[19] Ogata-Kawata H., Izumiya M., Kurioka D. (2014). Circulating exosomal microRNAs as biomarkers of colon cancer. *PLoS ONE*.

[20] Meng X., Müller V., Milde-Langosch K., Trillsch F., Pantel K., Schwarzenbach H. (2016). Diagnostic and prognostic relevance of circulating exosomal miR-373, miR-200a, miR-200b and miR-200c in patients with epithelial ovarian cancer. *Oncotarget*.

[21] Cha D. J., Franklin J. L., Dou Y. (2015). KRAS-dependent sorting of miRNA to exosomes. *eLife*.

[22] Ge Q., Zhou Y., Lu J., Bai Y., Xie X., Lu Z. (2014). MiRNA in plasma exosome is stable under different storage conditions. *Molecules*.

[23] Tian F., Shen Y., Chen Z., Li R., Lu J., Ge Q. (2016). Aberrant miR-181b-5p and miR-486-5p expression in serum and tissue of non-small cell lung cancer. *Gene*.

[24] Morrissy A. S., Morin R. D., Delaney A. (2009). Next-generation tag sequencing for cancer gene expression profiling. *Genome Research*.

[25] Livak K. J., Schmittgen T. D. (2001). Analysis of relative gene expression data using real-time quantitative PCR and the 2^−ΔΔCT^ method. *Methods*.

[26] Zavesky L., Jandakova E., Turyna R., Langmeierova L., Weinberger V., Minar L. (2016). Supernatant versus exosomal urinary microRNAs. Two fractions with different outcomes in gynaecological cancers. *Neoplasma*.

[27] Arroyo J. D., Chevillet J. R., Kroh E. M. (2011). Argonaute2 complexes carry a population of circulating microRNAs independent of vesicles in human plasma. *Proceedings of the National Academy of Sciences of the United States of America*.

[28] Chevillet J. R., Kang Q., Ruf I. K. (2014). Quantitative and stoichiometric analysis of the microRNA content of exosomes. *Proceedings of the National Academy of Sciences of the United States of America*.

[29] Joyce D. P., Kerin M. J., Dwyer R. M. (2016). Exosome-encapsulated microRNAs as circulating biomarkers for breast cancer. *International Journal of Cancer*.

[30] Chen W., Zheng R., Baade P. D. (2016). Cancer statistics in China, 2015. *CA: Cancer Journal for Clinicians*.

[31] Siegel R. L., Miller K. D., Jemal A. (2016). Cancer statistics, 2016. *CA: A Cancer Journal for Clinicians*.

[32] Skog J., Würdinger T., van Rijn S. (2008). Glioblastoma microvesicles transport RNA and proteins that promote tumour growth and provide diagnostic biomarkers. *Nature Cell Biology*.

[33] Kharaziha P., Ceder S., Li Q., Panaretakis T. (2012). Tumor cell-derived exosomes: a message in a bottle. *Biochimica et Biophysica Acta: Reviews on Cancer*.

[34] Hannafon B. N., Ding W.-Q. (2013). Intercellular communication by exosome-derived microRNAs in cancer. *International Journal of Molecular Sciences*.

