# Supplementary material for: No Significant Difference between Plasma miRNAs and Plasma-Derived Exosomal miRNAs from Healthy People
Source: Biomed Res Int. 2017 Jun 1;2017:1304816. doi: 10.1155/2017/1304816 (PMC5471588; doi:10.1155/2017/1304816)
Supplement: Supplementary file 1 — Supplemental Figure 1: The 12 differential miRNAs in the supernatant compared to plasma or exosome in the healthy group by sequencing. Supplemental Figure 2: The quantification results of 5 supernatant miRNAs in the healthy samples. Supplemental Table 1: The primer sequence used in the qRT-PCR assay. Supplemental Table 2: The differentially expressed miRNAs in plasma vs supernatant and in supernatant vs exosome by sequencing. [file 1304816.f1.pdf]

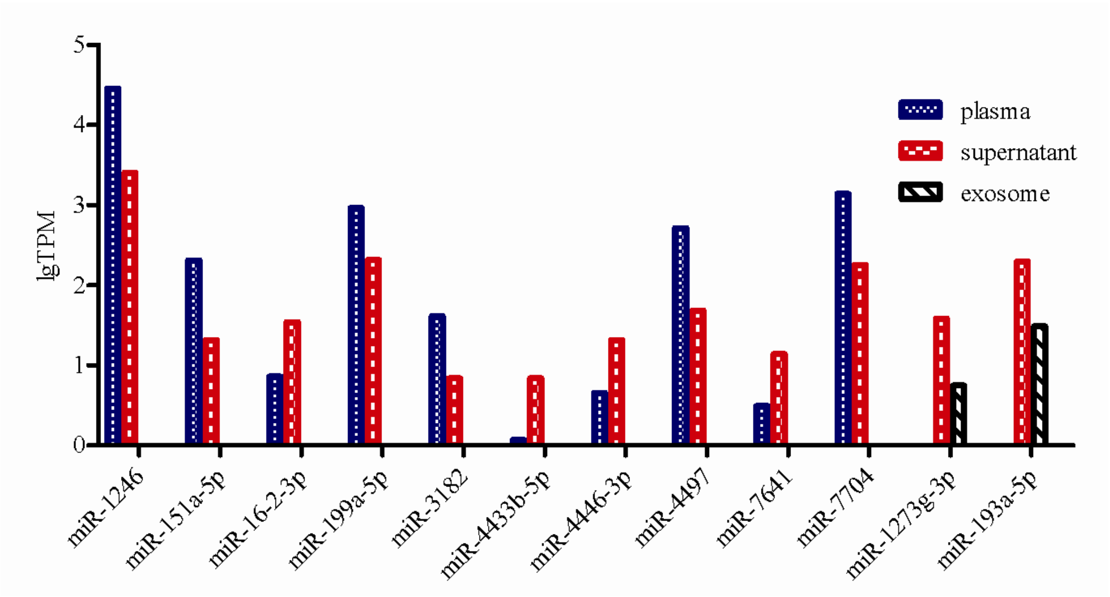

Supplemental Figure1 The 12 differential miRNAs in the supernatant compared to plasma or exosome in the healthy group by sequencing. The y-axis meant the value of TPM as log10. Ten miRNAs (6 up-regulated, 4 down-regulated) had significant differences between plasma and supernatant, and two miRNAs (up-regulated) were different in exosome compared to supernatant (p-value<0.05).

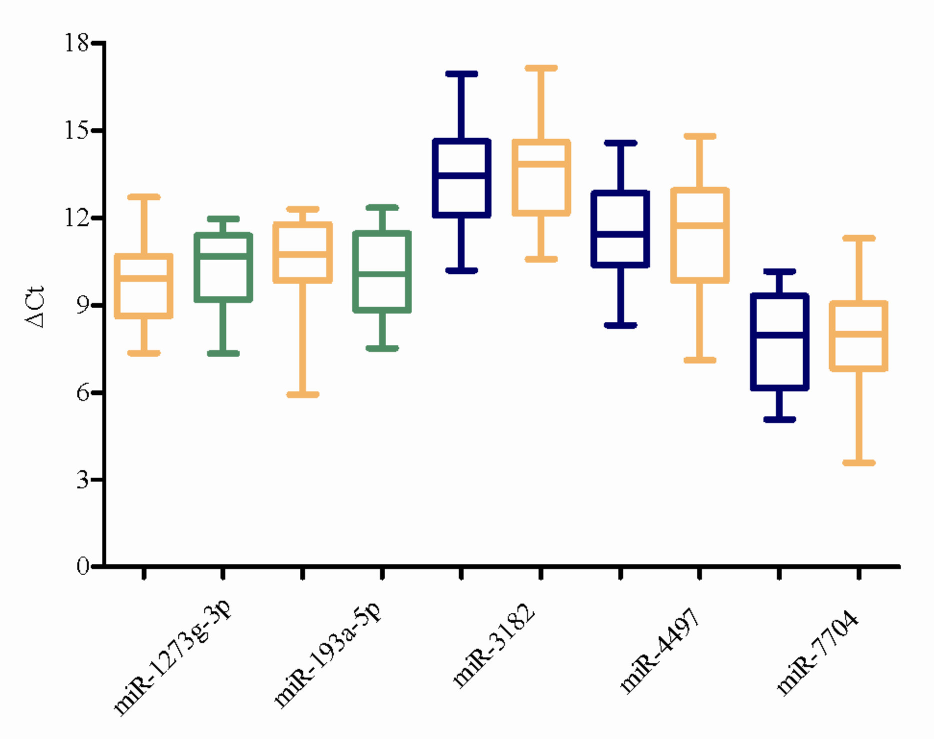

Supplemental Figure2 The quantification results of 5 supernatant miRNAs in the healthy samples. The supernatant miRNAs (yellow) did not have the differences when compared to plasma (blue) or exosome (green). The y-axis meant the value of $\Delta Ct = AvgCt_{miRNA} - AvgCt_{U6}$.

Supplemental Table1 The primer sequence used in the qRT-PCR assay

Primer Name	Sequence (5’-3’)
let-7a-5p RTP	GTCGTATCCAGTGCAGGGTCCGAGGTATTCGCACTGGATACGACAACTAT
miR-125a-5p RTP	GTCGTATCCAGTGCAGGGTCCGAGGTATTCGCACTGGATACGACTCACAG
miR-185-5p RTP	GTCGTATCCAGTGCAGGGTCCGAGGTATTCGCACTGGATACGACTCAGGA
miR-21-5p RTP	GTCGTATCCAGTGCAGGGTCCGAGGTATTCGCACTGGATACGACTCAACA
miR-423-3p RTP	GTCGTATCCAGTGCAGGGTCCGAGGTATTCGCACTGGATACGACACTGAG
miR-7704 RTP	GTCGTATCCAGTGCAGGGTCCGAGGTATTCGCACTGGATACGACCACGTC
miR-4497 RTP	GTCGTATCCAGTGCAGGGTCCGAGGTATTCGCACTGGATACGACGCCCAG
miR-3182 RTP	GTCGTATCCAGTGCAGGGTCCGAGGTATTCGCACTGGATACGACGACTAC
miR-193a-5p RTP	GTCGTATCCAGTGCAGGGTCCGAGGTATTCGCACTGGATACGACTCATCT
miR-1273g-3p RTP	GTCGTATCCAGTGCAGGGTCCGAGGTATTCGCACTGGATACGACCTCAGG
miR-144-5p RTP	GTCGTATCCAGTGCAGGGTCCGAGGTATTCGCACTGGATACGACCTTACA
miR-1-3p RTP	GTCGTATCCAGTGCAGGGTCCGAGGTATTCGCACTGGATACGACATACAT
miR-486-5p RTP	GTCGTATCCAGTGCAGGGTCCGAGGTATTCGCACTGGATACGACCTCGGG
miR-181b-5p RTP	GTCGTATCCAGTGCAGGGTCCGAGGTATTCGCACTGGATACGACACCCAC
Universal PCR Primer	GTGCAGGGTCCGAGGT
U6 RTP	GCTTCACGAATTTGCGTGTCAT
U6 F	GCTTCGGCAGCACATATACTAAA
U6 R	GCTTCACGAATTTGCGTGTCAT
let-7a-5p F	CGGGCTGAGGTAGTAGGTTGT
miR-125a-5p F	CCCTCCCTGAGACCCTTTAAC
miR-185-5p F	GGGTGGAGAGAAAGGCAGT
miR-21-5p F	CCGCCTAGCTTATCAGACTGA
miR-423-3p F	TTAGCTCGGTCTGAGGCCC
miR-7704 F	AATTACGGGGTCGGCGGC
miR-4497 F	AATTTGGCTCCGGGACGG
miR-3182 F	GTGGCCCGCTTCTGTAGT
miR-193a-5p F	AATGGGTCTTTGCGGGCG
miR-1273g-3p F	GTCCACCACTGCACTCCAG
miR-144-5p F	TTAGGATATCATCATATAC
miR-1-3p F	CCGTGGAATGTAAAGAAGT
miR-486-5p F	TCCTGTACTGAGCTGCCC
miR-181b-5p F	CAACATTCATTGCTGTCGGT

* RTP: reverse transcription primer; F: forward primer; R: reverse primer

Supplemental Table2 The differentially expressed miRNAs in plasma vs supernatant and in supernatant vs exosome by sequencing

	miRNA	log_2 (fold change)	p-value
plasma vs supernatant	hsa-miR-1246	3.48	0.0010
	hsa-miR-151a-5p	3.30	0.0207
	hsa-miR-16-2-3p	-2.25	0.0366
	hsa-miR-199a-5p	2.15	0.0157
	hsa-miR-3182	2.57	0.0005
	hsa-miR-4433b-5p	-2.56	0.0330
	hsa-miR-4446-3p	-2.20	0.0018
	hsa-miR-4497	3.41	0.0002
	hsa-miR-7641	-2.15	0.0006
	hsa-miR-7704	2.94	0.0009
supernatant vs exosome	hsa-miR-1273g-3p	2.78	0.0170
	hsa-miR-193a-5p	2.69	0.0215
